# Superior effect of allopurinol compared to febuxostat on the retardation of chronic kidney disease progression

**DOI:** 10.1371/journal.pone.0264627

**Published:** 2022-02-28

**Authors:** Seokwoo Park, Jung Pyo Lee, Dong Ki Kim, Yon Su Kim, Chun Soo Lim

**Affiliations:** 1 Department of Biomedical Sciences, Seoul National University College of Medicine, Seoul, Republic of Korea; 2 Department of Internal Medicine, Seoul National University College of Medicine, Seoul, Republic of Korea; 3 Department of Internal Medicine, Seoul National University Bundang Hospital, Seongnam, Korea; 4 Department of Internal Medicine, Seoul National University Boramae Medical Center, Seoul, Korea; 5 Department of Internal Medicine, Seoul National University Hospital, Seoul, Korea; Baker IDI Heart and Diabetes Institute, AUSTRALIA

## Abstract

**Background:**

Although hyperuricemia is associated with chronic kidney disease, whether and how it should be managed for renoprotection remains debatable. Thus, we investigated whether allopurinol and febuxostat, the most frequently used urate-lowering treatments, have differential renoprotective effects on chronic kidney disease.

**Methods:**

Incident users of allopurinol and febuxostat were identified from two tertiary referral centers. One-to-one propensity score matching between the allopurinol and febuxostat groups was performed. Participants were followed up until the occurrence of clinical outcomes, urate-lowering agent discontinuation, mortality, or the end of the study period, whichever occurred first. The primary outcomes were a 30% decline in estimated glomerular filtration rate (eGFR) and end-stage renal disease. Differential trends of eGFR decline were estimated using a linear mixed-effects model.

**Results:**

Each group included 654 participants. Baseline eGFRs were 40.1 [26.6–57.3] and 39.1 [27.9–58.3] mL/min/1.73 m^2^ in the allopurinol and febuxostat group, respectively. Adjusted least square mean change in serum urate was −1.58 mg/dL [95% confidence interval (CI), −1.78 to −1.38] and -2.69 mg/dL (95% CI, −2.89 to −2.49) in the allopurinol and febuxostat groups, respectively. Despite lower serum urate levels, febuxostat was significantly more associated with a 30% decline in eGFR (hazard ratio 1.26; 95% CI 1.03–1.54) and end-stage renal disease (hazard ratio 1.91, 95% CI 1.42–2.58) than allopurinol. Annual eGFR decline in febuxostat users was estimated to be more rapid than in allopurinol users by 2.14 (standard error 0.71) mL/min/1.73 m^2^ per year.

**Conclusions:**

Allopurinol demonstrated attenuation of chronic kidney disease progression and prevention of hypouricemia, compared to febuxostat. Because the treatment can be renoprotective, further studies on its effects on chronic kidney disease are required.

## Introduction

The association between hyperuricemia and chronic kidney disease (CKD) has long been recognized by nephrologists [1, 2]. Mechanistically, hyperuricemia activates the renin-angiotensin-aldosterone system, thus contributing to the development of systemic hypertension [3] and ensuing arteriolar diseases, resulting in reduced afferent autoregulation and glomerular hypertension [4]. Additionally, intracellular urate induces oxidative stress [5] leading to inflammatory responses [6]. Consistent with the mechanism studies, hyperuricemia predicts clinical outcomes of proteinuria [7], onset of CKD [1], and progression to end-stage renal disease (ESRD) [8].

Based on the potential etiological roles of hyperuricemia, many studies have been performed in an attempt to delay CKD progression by urate-lowering therapy (ULT), most commonly including allopurinol and febuxostat treatment [9]. Although several lines of evidence, such as randomized controlled trials, suggest that allopurinol could prevent the development [10] or progression of CKD [11, 12] contradictory results have hampered drawing a solid conclusion [9]. Febuxostat also showed inconsistent renoprotective effects in patients with CKD in randomized trials [13, 14]. Thus, whether and how ULT should be initiated for the sole purpose of retarding CKD progression is still debatable. In this regard, more research on patients with earlier stages of CKD and different ethnicities is required [9, 15]. Moreover, in designing such trials, which ULT might be the appropriate comparator to placebo is yet to be answered.

Allopurinol and febuxostat, both of which belong to the xanthine oxidase inhibitor category, are the most widely used urate-lowering agents. Although allopurinol has long been effective in mitigating hyperuricemia, it is associated with a risk of fatal hypersensitivity reactions [16]. In comparison with allopurinol, febuxostat is a more selective xanthine oxidase inhibitor and more potent in lowering serum urate levels [17]. However, given previous studies, determining which of these is better in terms of kidney protection is controversial. While allopurinol was associated with a lower risk of incident renal disease compared with febuxostat in a research [18], febuxostat seemed to be more renoprotective according to a small meta-analysis [19]. Hence, direct comparison of allopurinol and febuxostat regarding their effects on kidney function trajectory may provide useful information for designing future studies. Here, we investigated the association of renal function decline with the choice of ULT, either allopurinol or febuxostat, in incident users with CKD. In addition, the post-treatment serum urate levels and annual rates of eGFR decline under ULT were compared.

## Materials and methods

### Ethics statements

The study protocol complied with the Declaration of Helsinki and was approved by the Institutional Review Boards of the Seoul National University Hospital (H-1605-018-760) and the Seoul National University Boramae Medical Center (26-2016-46/042). The requirement for informed consent was waived by the board.

### Data sources and study population

We designed a multicenter retrospective cohort study using the electronic medical records of two tertiary referral centers, Seoul National University Hospital and Seoul National University Boramae Medical Center. Patients who had records of one or more prescriptions of allopurinol or febuxostat from January 1, 2011, to April 30, 2016, were identified. From the initial population, we excluded 8,520 participants based on the following exclusion criteria: [1] history of allopurinol or febuxostat prescription during the six months before the enrollment period; [2] younger than 18 years of age; [3] history of renal replacement therapy before enrollment; [4] treatment duration of less than three months; [5] follow-up records of less than three months; [6] less than 15 mL/min/1.73 m^2^ of baseline eGFR, and [7] prescription history of two or more types of ULTs during the study period. The baseline characteristics of the remaining participants were compared between allopurinol (n = 1,548) and febuxostat (n = 664) users. Due to significant between-group differences in the crude population, propensity scores were calculated. After one-to-one propensity matching, 634 participants in each group were included in subsequent analyses.

### Exposure ascertainment

Data on ULT prescriptions were obtained from the electronic medical records. The index date was designated as the day when the participants were first prescribed ULT during the enrollment period. Participants were allocated to the allopurinol or febuxostat groups based on the ULT prescribed on the index date. Discontinuation was defined as a grace period of more than 30 days until subsequent ULT prescription.

### Baseline data collection

Baseline characteristics were based on the values obtained on the index date. Comorbidities were defined by two or more records of diagnostic codes from the International Classification of Diseases, 10th revision for a previous year from the index date (S1 Table). Two or more incidences of prescription for a previous year were considered concomitant medication use at baseline. Missing values were imputed by a random forest algorithm using the missForest package (version 1.4).

### Measurements of serum urate and creatinine

Outpatient-based measurements of serum urate and creatinine levels for all enrolled participants were retrospectively obtained during the entire follow-up period. The kinetic alkaline picrate reaction (Jaffe) method was used to measure serum creatinine levels. Then, eGFR was calculated using the CKD-EPI equation and serum creatinine levels [20]. The following instruments were used to determine the laboratory values in this study: Hitachi Clinical Analyzer 747 (Hitachi Ltd., Tokyo, Japan), Toshiba 200FR (Toshiba Medical System Co., Tokyo, Japan), and Modular D2400 and ISE900 analyzers (Hitachi Ltd., Tokyo, Japan).

### Clinical outcomes

The primary outcomes were a 30% decline in eGFR [21] and ESRD. ESRD was defined as dialysis for over three months, or kidney transplantation. Sensitivity analyses were performed using 40% and 50% decline in eGFR as surrogate outcomes. Participants were followed up from the index date until the occurrence of clinical outcomes, ULT discontinuation, mortality, or the end of the study period, whichever occurred first.

### Statistical analyses

Baseline characteristics were expressed as mean ± standard deviation for normally distributed continuous variables and median with interquartile ranges for skewed variables. Normality was tested using the Shapiro-Wilk test. Categorical variables were described as counts with percentages. Before propensity matching, we compared between-group differences using the *t*-test or Mann-Whitney U test as appropriate for continuous variables, and chi-square or Fisher’s exact test for categorical variables. To deal with biases in baseline characteristics, we calculated the propensity scores of the treatment groups using multivariable logistic regression models, including all baseline variables [22]. One-to-one propensity score-based matching was performed using the nearest neighbor method. Standardized differences were estimated to test the balance after matching.

To compare the efficacies in lowering serum urate levels between the allopurinol and febuxostat groups, the interval change in serum urate from baseline to the last measurement was calculated. The least square mean and 95% confidence intervals (CIs) of the changes were estimated with adjustments for the time intervals [23].

The Kaplan-Meier method was used to describe event-free probabilities. The stratified log-rank test was used to compare survival functions. The risks of primary outcomes associated with the choice of ULTs were analyzed using stratified Cox proportional hazards regression. The hazard ratios (HRs) and 95% CIs were calculated. The proportional hazards assumption was evaluated using a log minus log plot.

A linear mixed-effects model was fitted to simulate annual eGFR decline from repeatedly measured values during the study period using the lme4 R package. Each participant was considered to have a random effect, and serum urate was considered a time-varying covariate. The interaction term between time and ULT was included in the regression model to assess the differential trends in eGFR decline depending on ULT. Random-intercept and random-slope models were used in this study.

Statistical significance was set at p<0.05 (two-tailed). Statistical analyses were performed using R software (version 4.1.2; R Foundation).

## Results

### Study population and baseline characteristics

**Fig 1:**
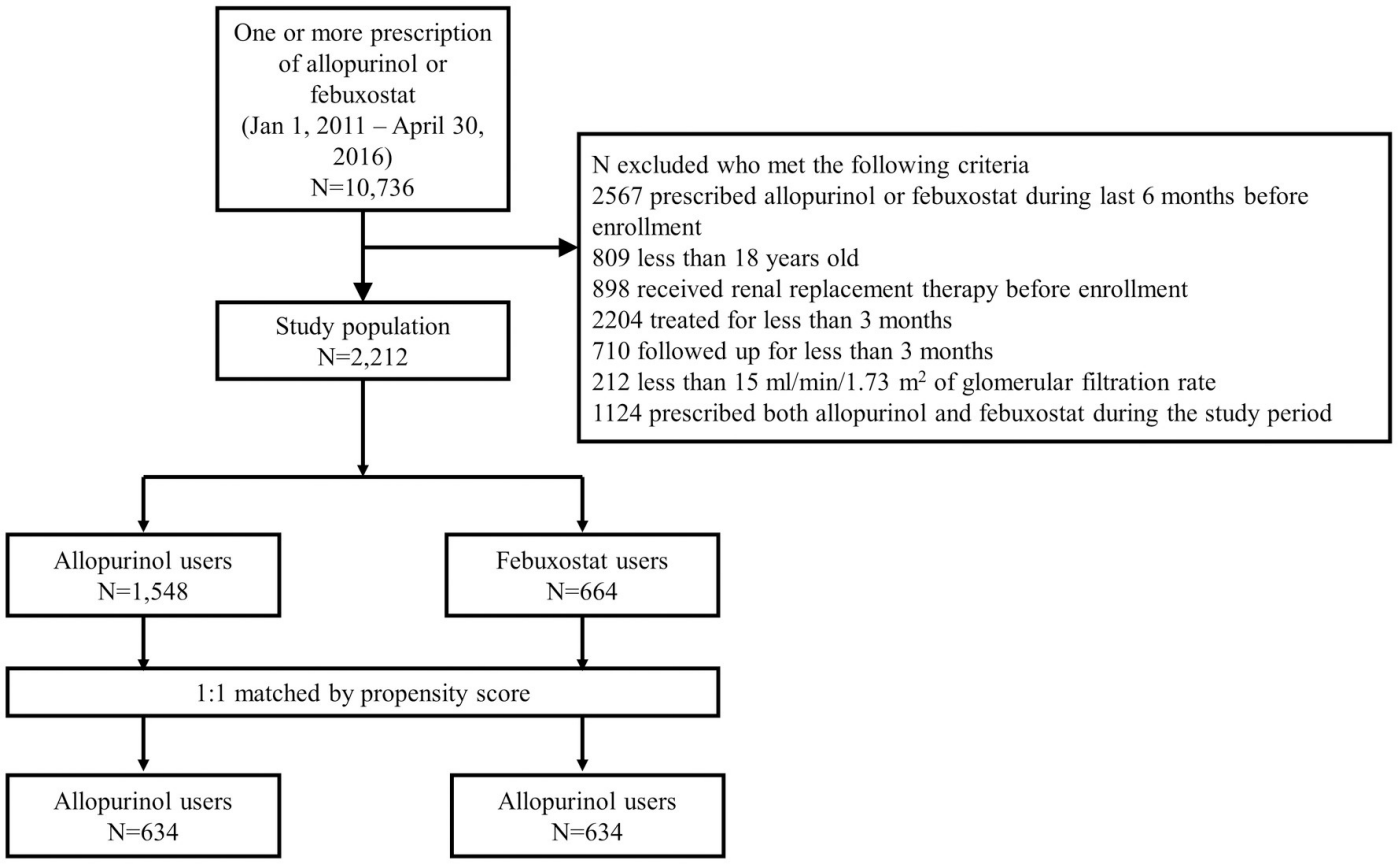
Flow diagram of the study.

We initially obtained 10,736 patients who were prescribed allopurinol or febuxostat between January 2011 and April 2016. After applying the pre-determined exclusion criteria, 2,212 participants were included in the study (S2 Table). Due to significant differences in baseline characteristics, we additionally performed 1:1 propensity score matching, resulting in 634 participants per group as the final study population (Fig 1).

Table 1 shows the baseline characteristics of the study population. With less than 10% standardized differences for all variables, the balance between the two groups was acceptable in the matched population. Mean eGFRs were 40.1 [26.6–57.3] mL/min/1.73 m^2^ and 39.1 [27.9–58.3] mL/min/1.73 m^2^ in the allopurinol and febuxostat group, respectively. Notably, approximately 20% of the patients were diabetic, and 60% were on angiotensin-converting enzyme inhibitor/angiotensin II receptor blockers. More than 15% of patients included in the study had gout.

**Table 1:** Baseline characteristics of the study population after missing value imputation and propensity score matching.

	After matching		
	Allopurinol	Febuxostat	Std. diff.^a^
	(n = 634)	(n = 634)	
Mean daily dose during the study period (mg/day)	100.0 [100.0–141.6]	40.0 [40.0–46.5]	
Age (years)	61.0 [50.0–72.0]	62.0 [48.0–73.0]	0.009
Male, n (%)	483 (76.2%)	487 (76.8%)	0.015
Body mass index (kg/m^2^)	24.8 [22.6–26.6]	24.7 [22.9–27.0]	0.004
Comorbidities, n (%)			
Gout	96 (15.1%)	103 (16.2%)	0.031
Diabetes	143 (22.6%)	142 (22.4%)	0.004
Hypertension	142 (22.4%)	134 (21.1%)	0.031
Dyslipidemia	97 (15.3%)	89 (14.0%)	0.036
Cerebrovascular disease	62 (9.8%)	59 (9.3%)	0.016
Ischemic heart disease	90 (14.2%)	95 (15.0%)	0.022
Heart failure	9 (1.4%)	16 (2.5%)	0.060
Peripheral vascular diseases	14 (2.2%)	15 (2.4%)	0.011
Liver cirrhosis	16 (2.5%)	15 (2.4%)	0.011
Medication, n (%)			
ACEi/ARB	393 (62.0%)	375 (59.1%)	0.058
Beta blocker	211 (33.3%)	216 (34.1%)	0.017
Calcium channel blocker	296 (46.7%)	294 (46.4%)	0.006
Statin	274 (43.2%)	269 (42.4%)	0.016
Thiazide	99 (15.6%)	102 (16.1%)	0.013
Loop diuretics	133 (21.0%)	154 (24.3%)	0.076
Colchicine	89 (14.0%)	91 (14.4%)	0.009
NSAID	101 (15.9%)	99 (15.6%)	0.009
Insulin	37 (5.8%)	43 (6.8%)	0.036
Serum urate (mg/dL)	8.3 [7.1–9.6]	8.7 [6.9–9.9]	0.015
eGFR (mL/min/1.73 m^2^)	40.1 [26.6–57.3]	39.1 [27.9–58.3]	0.020
eGFR stage^b^			
I	41 (6.5%)	44 (6.9%)	
II	101 (15.9%)	106 (16.7%)	
IIIa	122 (19.2%)	99 (15.6%)	
IIIb	176 (27.8%)	198 (31.2%)	
IV	194 (30.6%)	187 (29.5%)	
Spot urine protein-to-creatinine (g/g)	0.5 [0.2–1.5]	0.6 [0.2–1.5]	0.092
HDL (mg/dL)	47.6 [41.0–56.4]	48.0 [40.0–54.8]	0.014
LDL (mg/dL)	99.7 [85.0–113.8]	101.0 [85.2–117.2]	0.051
HbA1c (%)	6.1 [5.8–6.5]	6.1 [5.8–6.5]	0.024

Abbreviations: ACEi, angiotensin-converting enzyme inhibitor; ARB, angiotensin II receptor blocker; NSAID, non-steroidal anti-inflammatory drugs; eGFR, estimated glomerular filtration rate; HDL, high-density lipoprotein; LDL, low-density lipoprotein.

^a^Std. diff., standardized differences.

^b^Stage I, >= 90; II, < 90 and >= 60; IIIa, < 60 and >= 45; IIIb, <45 and >= 30; IV, <30 and >= 15 (ml/min/1.73 m^2^).

### Efficacies in lowering serum urate levels

The efficacy of allopurinol and febuxostat in lowering serum urate levels was estimated from interval changes during the follow-up period (Table 2). In the allopurinol group, the adjusted least square mean of change was −1.58 mg/dL (95% CI −1.78 to −1.38) from a baseline level of 8.4±2.1 mg/dL. The corresponding change in the febuxostat group was −2.69 mg/dL (95% CI −2.89 to −2.49) from a baseline level of 8.4±2.5 mg/dL. Allopurinol users demonstrated higher serum urate levels (6.7±1.6 mg/dL) measured at the end of the follow up than febuxostat users (5.8±2.2 mg/dL).

**Table 2:** Changes in serum urate during the follow-up periods of participants within a propensity score matched population.

Group	Serum urate (mg/dL)^a^		Interval change (mg/dL)	
	Baseline	Last measurements	Unadjusted mean (95% CI)	Adjusted LSM^b^ (95% CI)
Allopurinol	8.4±2.1	6.7±1.6	−1.67 (−1.87, −1.47)	−1.58 (−1.78, −1.38)
Febuxostat	8.4±2.5	5.8±2.2	−2.60 (−2.80, −2.40)	−2.69 (−2.89, −2.49)

Abbreviation: CI, confidence interval.

^a^Mean ± standard deviation.

^b^Least square mean adjusted for the interval between baseline and last measurements.

### Associations of urate-lowering agents with renal outcomes

**Fig 2:**
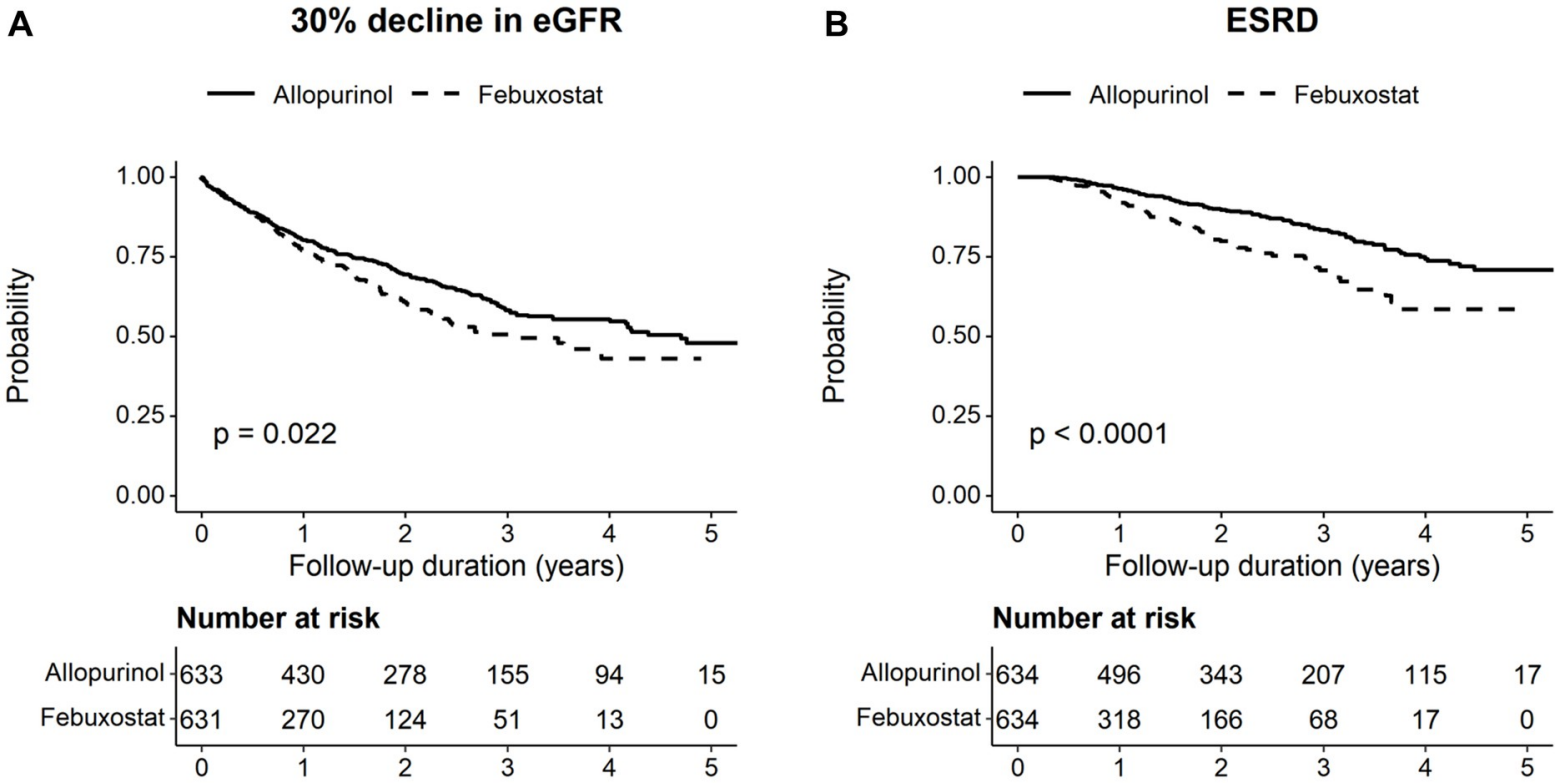
Kaplan-Meier curves for clinical outcomes according to the choice of urate-lowering agent. Event-free probabilities of (A) 30% decline in estimated glomerular filtration rate (eGFR) and (B) end-stage renal disease (ESRD).

The median follow-up durations were 411.0 days (2,054.3 person-years in total) for a 30% decline in eGFR and 552.0 days (2405.0 person-years in total) for ESRD. Among 1,268 participants, 407 experienced a 30% decline in eGFR and 181 progressed to ESRD. The incidence of the 30% decline in eGFR in allopurinol users (17.3 per 100 person-years; 95% CI 15.0–19.6) was lower than that in febuxostat users (23.9 per 100 person-years; 95% CI 20.5–27.3) (Table 3). In addition, allopurinol users (6.1 per 100 person-years; 95% CI 4.9–7.4) experienced less progression to ESRD than febuxostat users (9.8 per 100 person-years; 95% CI 7.8–11.9). Fig 2 illustrates the event-free probabilities of a 30% decline in eGFR and ESRD for both treatment groups. Allopurinol conferred significantly more benefit on event-free probabilities than febuxostat for both eGFR decline (*P* = 0.022) and ESRD (*P*<0.001). In Cox regression analyses, febuxostat was significantly more associated with a 30% decline in eGFR (HR 1.26; 95% CI 1.03–1.54) and ESRD (HR 1.91, 95% CI 1.42–2.58) compared to allopurinol.

**Table 3:** Incidences and associations of renal outcomes according to uric acid-lowering agent.

Renal outcome	Incidences (95% CI)^a^			HR (95% CI)	*p*-value
	Total	Allopurinol	Febuxostat	Febuxostat (versus allopurinol)	
30% decline in eGFR	19.8 (17.9–21.7)	17.3 (15.0–19.6)	23.9 (20.5–27.3)	1.26 (1.03–1.54)	0.022
End-stage renal disease	7.5 (6.4–8.6)	6.1 (4.9–7.4)	9.8 (7.8–11.9)	1.91 (1.42–2.58)	<0.001

Abbreviations: CI, confidence interval; eGFR, estimated glomerular filtration rate; HR, hazard ratio.

^a^ per 100 person-years.

Sensitivity analyses for 40% and 50% decline in eGFR consistently demonstrated a significantly higher risk of renal outcomes in the febuxostat group (S3 Table).

To investigate the effectiveness of the ULTs more specifically in CKD patients with decreased eGFR, we performed independent analyses within subgroups of baseline eGFR lower than 60 and 45 ml/min/1.73 m^2^. When we repeated the Cox regression analyses, febuxostat showed even larger effect sizes on renal outcomes, including eGFR decline and ESRD, compared with the original analyses on the entire matched population (S4 Table).

### Differential trends in the estimated glomerular filtration rate decline between urate-lowering agents

To further evaluate the effect of urate-lowering agents on eGFR change, we fitted a linear mixed-effects model for eGFR (Table 4). For simplicity, we hypothesized that eGFR declined linearly during the study period. The annual eGFR decline was estimated to be 2.08 (standard error [SE] 0.46) mL/min/1.73 m^2^ in the base case of the study population. Importantly, febuxostat users showed more rapid deterioration of estimated renal function than allopurinol users by 2.14 (SE 0.71) mL/min/1.73 m^2^ per year. Along with albuminuria, serum urate was a significant risk factor for decreased eGFR, which induced 1.52 (SE 0.04) mL/min/1.73 m^2^ of lower eGFR level per 1 mg/dL.

**Table 4:** Difference in annual eGFR decline depending on urate-lowering agents and other significant predictors for eGFR in linear mixed-effects model.

Variables^a^	Estimates (Standard error) (mL/min/1.73 m^2^)	*p*-value
Year* febuxostat (versus allopurinol)	−2.14 (0.71)	0.003
Year	−2.08 (0.46)	<0.001
eGFR at baseline (per 1 mL/min/1.73 m^2^)	0.93 (0.01)	<0.001
Serum urate (per 1 mg/dL)	−1.52 (0.04)	<0.001
Febuxostat (versus allopurinol)	−1.70 (0.57)	0.003
Urine protein (per 1 g/g urine creatinine)	−0.91 (0.14)	<0.001
Age (per 1 year of age)	−0.10 (0.02)	<0.001

Abbreviation: eGFR, estimated glomerular filtration rate.

^a^Listed in descending order of effect size.

## Discussion

In this study, we found that the risks of decline in kidney function and progression to ESRD were lower in the allopurinol group than in the febuxostat group among incident ULT users. This result was supported by a more rapid eGFR decline associated with febuxostat in linear mixed-effects model regression. Because it is not believed that febuxostat deteriorates kidney diseases more than placebo, we inferred that allopurinol may have a renoprotective role. The protective effect of allopurinol could not be completely explained by its ability to lower serum urate levels because febuxostat was more effective in reducing serum urate levels.

The benefit of allopurinol on CKD progression in our study was largely consistent with the results of previous clinical studies [11, 12, 24, 25]. Importantly, the most recent placebo-controlled trials, Controlled Trial of Slowing of Kidney Disease Progression from the Inhibition of Xanthine Oxidase (CKD-FIX) [26] and Preventing Early Renal Loss in Diabetes (PERL) [27] however, have failed to demonstrate better renal outcomes in the allopurinol group compared with placebo. One explanation for the inconsistent results is that in the CKD-FIX and PERL trials, participants with established CKD were recruited, limiting the generalizability to young and early stage patients [15]. In addition, Sato et al. previously revealed that in most randomized controlled trials with negative results, the absolute rates of CKD progression in the control groups were clinically irrelevant, these were defined as an eGFR decline of <4 mL/min/1.73 m^2^ over the study period (median, 12 months; range, 3–84 months) [9]. Therefore, the treatment effects of allopurinol can be appropriately evaluated in patients who are subject to significant deterioration. In our study, participants in the febuxostat group, which served as control against the allopurinol group, showed an annual eGFR decline of 3.84 mL/min/1.73 m^2^ in the linear mixed-effects model.

The relative benefit of allopurinol compared to febuxostat is a novel finding with respect to the retardation of CKD progression, although it has been reported to prevent incident kidney disease [18]. Notably, this contrasts with a few previous reports describing the superior renoprotective effect of febuxostat in CKD compared to allopurinol. However, several of these studies had small sample sizes [28, 29]. Another major caveat of other studies was a short median follow-up duration of less than one year [28, 30]. Because allopurinol can cause acute reduction in GFR due to reduced glomerular pressure [4], a sufficient study duration is required to adequately capture its effect on CKD. Similarly, early CKD patients may become a more appropriate target population to avoid unintended adverse events from acute hemodynamic effects. For example, in a recent study of a CKD cohort from Taiwan, allopurinol did not significantly differ from febuxostat in reducing the risk of progression to ESRD [23]. Nevertheless, we infer that a smaller sample size, shorter follow-up duration, and more advanced baseline renal dysfunction in the participants of the study may have contributed to the discrepancy from our study.

Febuxostat is considered a more potent urate-lowering agent than allopurinol [17]. Interestingly, despite the lower serum urate level after treatment in the febuxostat group, more renal outcomes were prevented in the allopurinol group in our study. There are possible explanations for this finding. First, as allopurinol is a less specific purine analog than febuxostat, off-target or pleiotropic effects of allopurinol aside from serum urate reduction can be present [31]. In support of this, allopurinol was reported to increase ATP flux [31] and prevent oxidative stress in glomerular endothelial cells [32]. Second, low serum urate could contribute to the loss of kidney function in the febuxostat group [33]. Considering the mean and standard deviation of serum urate in the febuxostat group after treatment, a significant proportion of patients may have experienced mild hypouricemia (<5 mg/dL) during the study period. Based on the results of our study, we suggest that excessive correction of hyperuricemia should be avoided, because serum urate level may have a U-shaped association with renal outcomes. Third, organ-specific effects of the two drugs may have been present. Furthermore, febuxostat is still suspected to induce more cardiovascular risks than allopurinol, regardless of serum urate level [34]. Hence, cardiovascular dysfunction could be an aggravating factor of CKD in the febuxostat group.

This study has several limitations. First, selection biases may have confounded the study results because of the retrospective design. We addressed this issue by propensity matching, and the final study population showed well-balanced baseline characteristics. Because long-term follow-up data of renal trajectories under ULTs are scarce, especially in a broad range of CKD patients, observations made using real-world data are valuable. Second, the study drugs were not compared with placebo or no treatment control. Recent meta-analyses have reported conflicting results regarding the efficacy of ULTs in renal outcomes [35–37]. Thus, the results of our study should not be interpreted as direct evidence of renal protection by allopurinol, but rather as the relative preference for allopurinol over febuxostat in hyperuricemic patients. Additionally, the study provides a formal rationale for selecting allopurinol instead of febuxostat as a comparator to placebo in future trials. Third, the linear trend we assumed to simulate eGFR decline may not reflect the true trajectories of disease progression [38]. However, deviations derived from the linearity assumption may not be substantial within our study duration, although long-term trajectories would be non-linear. Moreover, the estimates obtained in the linear model are straightforward and correlate with real data.

## Conclusions

We revealed that allopurinol is superior to febuxostat in patients with CKD for the protection of renal function. Because the risks of serious adverse events from allopurinol treatment did not differ from placebo in recent large randomized controlled trials [26, 27], we agree with the recommendation that allopurinol is the first drug of choice for the treatment of hyperuricemia [9], unless it is uncontrollable despite optimal dose adjustment. Although further evidence is required, lowering serum urate with allopurinol, while avoiding hypouricemia, may delay CKD progression, especially in early stage patients. Considering the potential benefits of allopurinol, further well-designed studies are needed to investigate the impact of allopurinol on CKD.

## Supporting information

S1 Table Diagnostic codes from the International Classification of Diseases, 10th revision (ICD-10) used for definition of covariates. (DOCX)

S2 Table Baseline characteristics of the study population before propensity score matching. (DOCX)

S3 Table Incidences and associations of renal outcomes according to uric acid-lowering agents. (DOCX)

S4 Table Incidences and associations of renal outcomes according to uric acid-lowering agents in subgroups with lower baseline estimated glomerular filtration rates. (DOCX)
