# Supplementary material for: Superior effect of allopurinol compared to febuxostat on the retardation of chronic kidney disease progression
Source: PLoS One. 2022 Feb 28;17(2):e0264627. doi: 10.1371/journal.pone.0264627 (PMC8884483; doi:10.1371/journal.pone.0264627)
Supplement: S1 Table — (DOCX) [file pone.0264627.s001.docx]

**S1 Table. Diagnostic codes from the International Classification of Diseases, 10th revision (ICD-10) used for definition of covariates.**

| **Variable** | **ICD-10 codes** |
| --- | --- |
| Gout | M10 |
| Diabetes | E10.9, E11.9, E13.9, E14.9, E10.1, E11.1, E13.1, E14.1, E10.5, E11.5, E13.5, E14.5 |
| Hypertension | I10-15 |
| Dyslipidemia | E78 |
| Cerebrovascular disease | I60-66, G45.0-45.2, G45.8, G45.9, G46, G45.4, I67.0-67.2, I67.4-67.9, I68.1, I68.2, I68.8, I69 |
| Ischemic heart disease | I20-25 |
| Heart failure | I50 |
| Peripheral vascular disease | I71, I79.0, I73.9, R02, Z95.8, Z95.9 |
| Liver cirrhosis | K70.3, K74.3-74.6 |
