# Supplementary material for: Superior effect of allopurinol compared to febuxostat on the retardation of chronic kidney disease progression
Source: PLoS One. 2022 Feb 28;17(2):e0264627. doi: 10.1371/journal.pone.0264627 (PMC8884483; doi:10.1371/journal.pone.0264627)
Supplement: S2 Table — (DOCX) [file pone.0264627.s002.docx]

**S2 Table. Baseline characteristics of the study population before propensity score matching.**

|  | Before matching | | | Number of cases with missing values | |
| --- | --- | --- | --- | --- | --- |
|  | Allopurinol  (n=1,548) | Febuxostat  (n=664) | *P*-value | Allopurinol | Febuxostat |
| Age (years) | 58.0 [45.0-69.0] | 62.0 [48.0-73.0] | <.001 |  |  |
| Male, n (%) | 1,207 (78.0%) | 510 (76.8%) | 0.585 |  |  |
| Body mass index (kg/m^2^) | 24.1 [21.8-26.4] | 24.4 [21.8-26.5] | 0.566 | 483 (31.2%) | 171 (25.8%) |
| Comorbidities, n (%) |  |  |  |  |  |
| Gout | 344 (22.2%) | 103 (15.5%) | <.001 |  |  |
| Diabetes | 231 (14.9%) | 162 (24.4%) | <.001 |  |  |
| Hypertension | 343 (22.2%) | 142 (21.4%) | 0.729 |  |  |
| Dyslipidemia | 236 (15.2%) | 94 (14.2%) | 0.553 |  |  |
| Cerebrovascular disease | 131 (8.5%) | 62 (9.3%) | 0.558 |  |  |
| Ischemic heart disease | 153 (9.9%) | 105 (15.8%) | <.001 |  |  |
| Heart failure | 9 (0.6%) | 23 (3.5%) | <.001 |  |  |
| Peripheral vascular diseases | 20 (1.3%) | 15 (2.3%) | 0.138 |  |  |
| Liver cirrhosis | 33 (2.1%) | 15 (2.3%) | 0.977 |  |  |
| Medication, n (%) |  |  |  |  |  |
| ACEi/ARB | 702 (45.3%) | 400 (60.2%) | <.001 |  |  |
| Beta blocker | 349 (22.5%) | 236 (35.5%) | <.001 |  |  |
| Calcium channel blocker | 494 (31.9%) | 315 (47.4%) | <.001 |  |  |
| Statin | 490 (31.7%) | 295 (44.4%) | <.001 |  |  |
| Thiazide | 206 (13.3%) | 108 (16.3%) | 0.078 |  |  |
| Loop diuretics | 229 (14.8%) | 172 (25.9%) | <.001 |  |  |
| Colchicine | 221 (14.3%) | 91 (13.7%) | 0.774 |  |  |
| NSAID | 309 (20.0%) | 102 (15.4%) | 0.013 |  |  |
| Insulin | 47 (3.0%) | 49 (7.4%) | <.001 |  |  |
| Serum urate | 8.0 [6.5-9.2] | 8.8 [7.2-9.9] | <.001 |  |  |
| eGFR (mL/min/1.73 m^2^) | 61.4 [40.6-85.9] | 37.9 [26.6-57.0] | <.001 |  |  |
| eGFR stage^a^ |  |  | <.001 |  |  |
| I | 317 (20.5%) | 44 (6.6%) |  |  |  |
| II | 485 (31.3%) | 106 (16.0%) |  |  |  |
| IIIa | 274 (17.7%) | 99 (14.9%) |  |  |  |
| IIIb | 262 (16.9%) | 206 (31.0%) |  |  |  |
| IV | 210 (13.6%) | 209 (31.5%) |  |  |  |
| Spot urine protein-to-creatinine (g/g) | 0.4 [0.1-1.2] | 0.8 [0.2-2.0] | <.001 | 811 (52.4%) | 141 (21.2%) |
| HDL (mg/dL) | 46.0 [39.0-57.5] | 46.0 [37.0-57.0] | 0.154 | 608 (39.3%) | 161 (24.2%) |
| LDL (mg/dL) | 96.0 [77.0-119.0] | 95.0 [73.0-118.0] | 0.443 | 742 (47.9%) | 234 (35.2%) |
| HbA1c (%) | 6.0 [5.6-6.7] | 6.2 [5.6-7.1] | 0.012 | 778 (50.3%) | 284 (42.8%) |

Abbreviations: ACEi, angiotensin converting enzyme inhibitor; ARB, angiotensin II receptor blocker; NSAID, non-steroidal anti-inflammatory drugs; eGFR, estimated glomerular filtration rate; HDL, high-density lipoprotein; LDL, low-density lipoprotein. ^a^Stage I, >= 90; II, < 90 and >= 60; IIIa, < 60 and >= 45; IIIb, <45 and >= 30; IV, <30 and >= 15 (ml/min/1.73m^2^)
