# Supplementary material for: Superior effect of allopurinol compared to febuxostat on the retardation of chronic kidney disease progression
Source: PLoS One. 2022 Feb 28;17(2):e0264627. doi: 10.1371/journal.pone.0264627 (PMC8884483; doi:10.1371/journal.pone.0264627)
Supplement: S3 Table — (DOCX) [file pone.0264627.s003.docx]

**S3 Table. Incidences and associations of renal outcomes according to uric acid-lowering agents.**

|  | Incidences (95% CI)^a^ | | | HR (95% CI) | *p*-value |
| --- | --- | --- | --- | --- | --- |
| Renal outcome | Total | Allopurinol | Febuxostat | Febuxostat (versus allopurinol) | |
| 40% decline in eGFR | 13.0 (11.5-14.5) | 11.5 (9.7–13.3) | 15.4 (12.8–18.0) | 1.29 (1.02–1.64) | 0.033 |
| 50% decline in eGFR | 8.8 (7.6–10.0) | 7.5 (6.1–8.9) | 10.9 (8.7–13.1) | 1.49 (1.13–1.97) | 0.005 |

Abbreviations: CI, confidence interval; eGFR, estimated glomerular filtration rate; HR, hazard ratio.

^a^ per 100 person-years.
