# Supplementary material for: Superior effect of allopurinol compared to febuxostat on the retardation of chronic kidney disease progression
Source: PLoS One. 2022 Feb 28;17(2):e0264627. doi: 10.1371/journal.pone.0264627 (PMC8884483; doi:10.1371/journal.pone.0264627)
Supplement: S4 Table — (DOCX) [file pone.0264627.s004.docx]

**S4 Table. Incidences and associations of renal outcomes according to uric acid-lowering agents in subgroups of lower baseline estimated glomerular filtration rates.**

|  | Number of subjects | Incidences (95% CI)^a^ | | | HR (95% CI) | p-value |
| --- | --- | --- | --- | --- | --- | --- |
|  |  | Total | Allopurinol | Febuxostat | Febuxostat (versus allopurinol) | |
| eGFR <60 (mL/min/1.73 m²) | 950 |  |  |  |  |  |
| 30% decline in eGFR |  | 23.9 (21.4-26.3) | 20.6 (17.6-23.5) | 29.3 (24.8-33.7) | 1.35 (1.09-1.67) | 0.006 |
| 40% decline in eGFR |  | 15.7 (13.7-17.6) | 13.4 (11.1-15.6) | 19.5 (16.0-23.0) | 1.45 (1.13-1.87) | 0.003 |
| 50% decline in eGFR |  | 10.4 (8.8-11.9) | 8.3 (6.6-10.0) | 13.8 (10.9-16.7) | 1.80 (1.33-2.43) | <0.001 |
| End-stage renal disease |  | 10.0 (8.5-11.5) | 8.2 (6.5-9.9) | 12.9 (10.2-15.6) | 1.90 (1.40-2.57) | <0.001 |
| eGFR <45 (mL/min/1.73 m²) | 740 |  |  |  |  |  |
| 30% decline in eGFR |  | 25.5 (22.6-28.4) | 20.9 (17.6-24.3) | 33.2 (27.7-38.6) | 1.51 (1.20-1.91) | 0.001 |
| 40% decline in eGFR |  | 17.2 (14.9-19.5) | 14.3 (11.7-16.9) | 22.1 (17.9-26.3) | 1.58 (1.20-2.07) | 0.001 |
| 50% decline in eGFR |  | 11.5 (9.7-13.3) | 8.7 (6.7-10.7) | 16.1 (12.6-19.6) | 2.00 (1.45-2.76) | 0.001 |
| End-stage renal disease |  | 11.5 (9.7-13.3) | 9.3 (7.2-11.3) | 15.1 (11.8-18.5) | 2.02 (1.47-2.78) | <0.001 |

Abbreviations: CI, confidence interval; eGFR, estimated glomerular filtration rate; HR, hazard ratio.

^a^ per 100 person-years.
